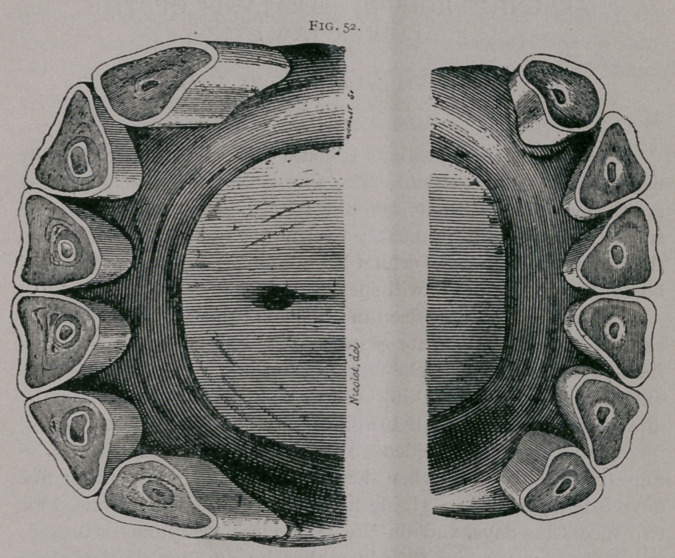# Age of the Horse, Ox, Dog and Other Domesticated Animals

**Published:** 1891-01

**Authors:** R. S. Huidekoper

**Affiliations:** Veterinarian


### AGE OF THE HORSE, OX, DOG, AND OTHER DOMESTICATED ANIMALS.

BY R. S. HUIDEKOPER, M.D., VETERINARIAN.

[*Continued from page 709, Vol. XI.*]

*Fifteen Years.*—FIG. 51.

From in front, the inferior teeth appear shorter than the superior, as the jaw has not been raised to the proper height; in profile, they are seen to be about the same length. The notch in the superior corner teeth continues. The inferior tables show in

FIG. 51.

Fifteen Years.

FIG. 51.

their centre a very decided, rounded dental star ; the pinchers are nearly triangular in shape ; the intermediate teeth begin to become so. The central enamel in the upper pinchers are much smaller than at thirteen years ; the incisive arch is depressed in front and narrowed transversely.

FIG. 52.

Seventeen Years.

FIG. 52.

*Seventeen Years.*—FIG. 52.

In front, the superior corner teeth incline toward the centre. The line of apposition of the teeth is very oblique on the horizontal line. To see the inferior teeth distinctly, the head must be well lifted. The tables of the inferior teeth are all triangular; the dental star is in the centre and distinctly round. The inferior incisive arch is narrow, only slightly convex, and the teeth appear more separated from each other than at any previous period; the pinchers especially are separated from each other in the median line. The tables of the superior teeth are triangular; the cup of the pinchers has nearly disappeared.

[TO BE CONTINUED.]

## Figures and Tables

**Fig. 51. f1:**
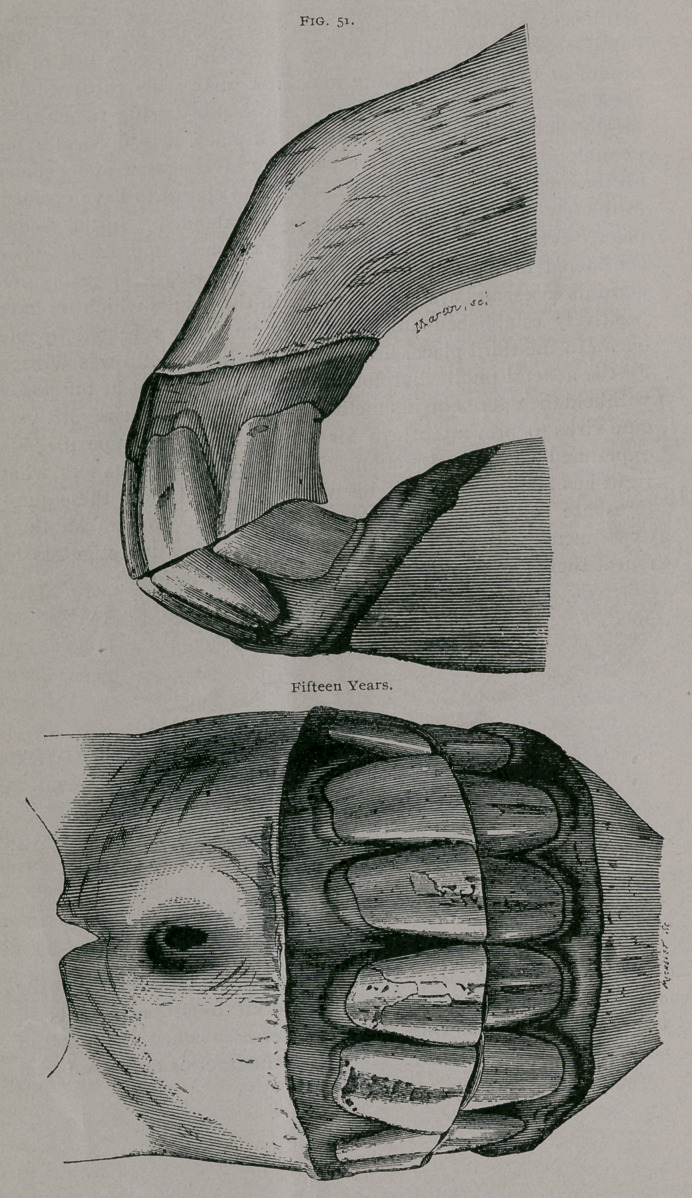


**Fig. 51. f2:**
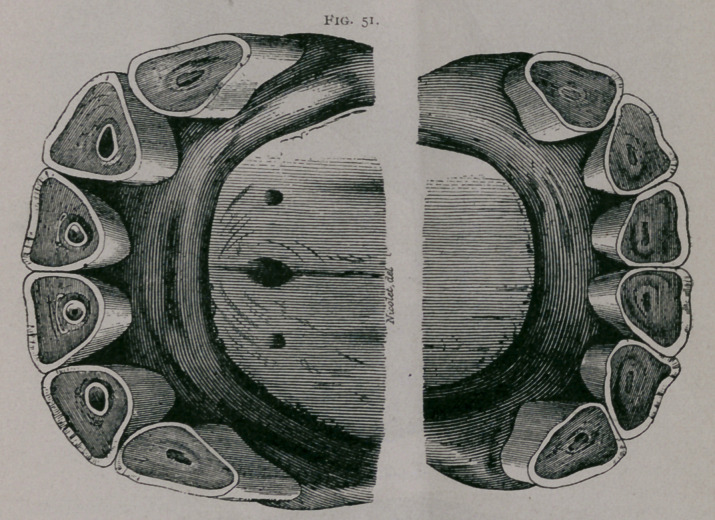


**Fig. 52. f3:**
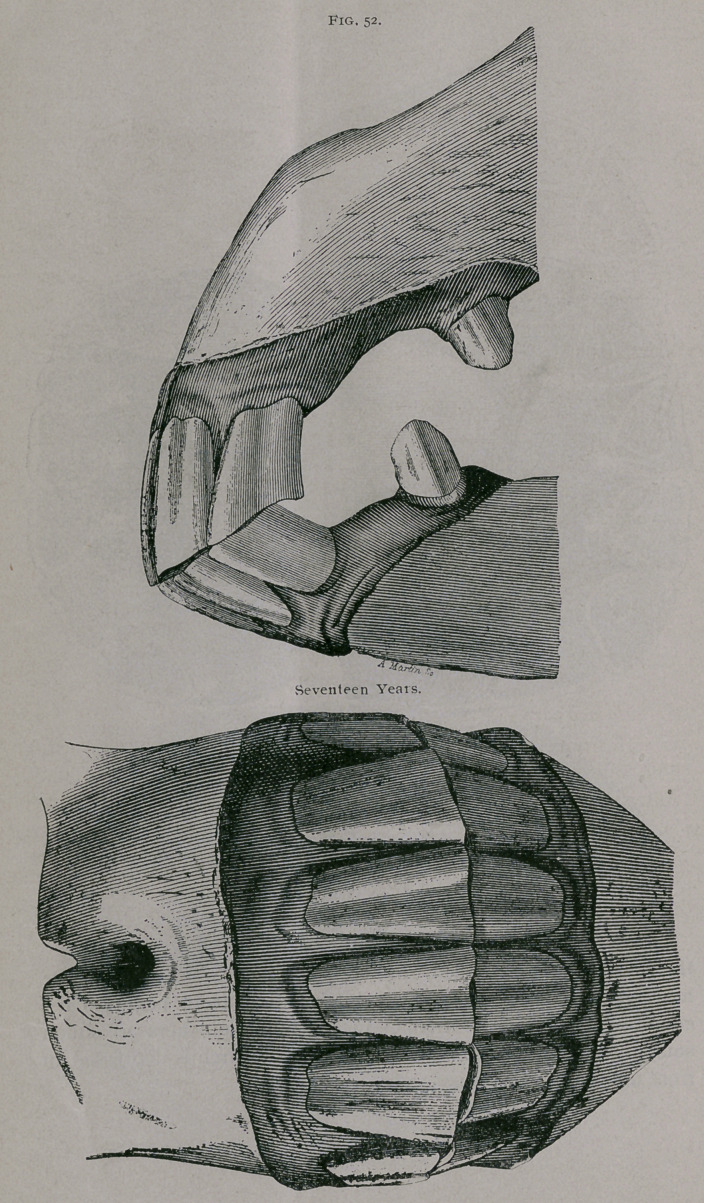


**Fig. 52. f4:**